# Increased Risks of Mortality and Atherosclerotic Complications in Incident Hemodialysis Patients Subsequently with Bone Fractures: A Nationwide Case-Matched Cohort Study

**DOI:** 10.1371/journal.pone.0121705

**Published:** 2015-04-13

**Authors:** Chiu-Huang Kuo, Tsung-Cheng Hsieh, Chih-Hsien Wang, Chu-Lin Chou, Yu-Hsien Lai, Yi-Ya Chen, Yu-Li Lin, Sheng-Teng Wu, Te-Chao Fang

**Affiliations:** 1 Division of Nephrology, Department of Internal Medicine, Buddhist Tzu Chi General Hospital, Hualien, Taiwan; 2 Institute of Medical Sciences, Tzu Chi University, Hualien, Taiwan; 3 Division of Nephrology, Department of Internal Medicine, Wan Fang Hospital, Taipei Medical University, Taipei, Taiwan; 4 Department of Internal Medicine, School of Medicine, College of Medicine, Taipei Medical University, Taipei, Taiwan; Universidade de São Paulo, BRAZIL

## Abstract

**Background:**

Hemodialysis (HD) patients with bone fractures have an increased risk for death. However, the risks for mortality and atherosclerotic complications in incident HD patients subsequently with bone fractures are unknown.

**Methods:**

Data derived from the Taiwan National Health Institute Research Database between January 1997 and December 2008 was analyzed. The enrolled patients included 3,008 incident HD patients subsequently with a single long bone fracture (LB Fx) and 2,070 incident HD patients subsequently with a single non-long bone fracture (NLB Fx). These patients were matched (1:5 ratio) for age, sex, and same duration of HD with incident HD patients who had no fractures and outcomes were measured over a 3-year follow-up.

**Results:**

After demographic and co-morbidity adjustment, LB Fx increased the risk for overall mortality (HR = 1.59, *p* < 0.001) and stroke (HR = 1.09, *p* = 0.028) in incident HD patients. NLB Fx increased the risk for overall mortality (HR = 1.52, *p* < 0.001), stroke (HR = 1.19, *p* < 0.001), coronary artery disease (CAD), (HR = 1.13, *p* = 0.003), and peripheral arterial occlusive disease (PAOD), (HR = 1.41, *p* < 0.001) in incident HD patients. Moreover, incident patients subsequently with NLB Fx had significantly higher risks of CAD and PAOD than those subsequently with LB Fx.

**Conclusions:**

The rates of mortality and stroke were significantly higher in incident HD patients subsequently with bone fractures than in matched patients without bone fractures. Incident HD patients subsequently with NLB Fx had significantly higher risks of CAD and PAOD than those subsequently with LB Fx and without bone fractures. Thus, incident HD patients subsequently with bone fractures should be closely followed for a higher mortality and possible development of atherosclerotic complications.

## Introduction

Previous studies of general populations reported significant associations between bone fracture and mortality [1], risk of second fracture [2,3], and cardiovascular death [4]. Dialysis patients have an increased incidence of all kinds of fractures compared to the general population [5]. Although there have been improvements in the management of mineral bone disorders, dialysis patients have still had a high incidence of fractures in the past decade [6,7]. In particular, bone fracture is one of the most common non-cardiovascular complications of end stage renal disease (ESRD) [6]. Previous observational studies showed that the risks of mortality and hospitalization were higher in dialysis patients with hip fractures [6,8], vertebral fractures [9], and all kinds of bone fractures [7,10]. A comparison of the different types of fractures indicated that vertebral fractures had the highest post-discharge mortality rate, re-hospitalization rate, and duration of hospitalization [7].

Chronic kidney disease-mineral bone disorder (CKD-MBD), which is defined by the presence of biochemical abnormalities, skeletal system abnormalities, and extra-skeletal calcification [11], is associated with increased cardiovascular disease and fractures in dialysis patients. The presentation of a CKD patient with cardiovascular events and fractures indicates CKD-MBD, and the presence of this disorder affects the mortality. However, the risk of atherosclerotic complications (stroke, coronary artery disease [CAD], acute coronary syndrome [ACS], peripheral artery occlusive diseases [PAOD]) in incident hemodialysis (HD) patients subsequently with bone fractures has not been evaluated. Moreover, there have been no comparisons of the atherosclerotic complications of incident HD patients subsequently with long bone fractures (LB Fx) *vs*. non-long bone fractures (NLB Fx).

In 1995, the Taiwan government established the National Health Insurance (NHI) program to provide compulsory universal health insurance. Almost all citizens of Taiwan are enrolled in the NHI. The data from the NHI is ideal for large longitudinal cohort studies [12–17]. Here, we used the NHI research database from 1997 to 2008 to compare the mortality and atherosclerotic outcomes of incident HD patients subsequently with LB Fx, NLB Fx, and no bone fractures.

## Materials and Methods

### Data sources

The data in this study was extracted from the National Health Institute Research Database (NHIRD) of Taiwan from January 1997 to December 2008. The NHIRD includes detailed information about health insurance, which covers 99% of the Taiwan population. A subgroup under the NHIRD is the Registry of Catastrophic Illnesses, which includes subjects with ESRD who underwent HD (our study subjects) and subjects with cancer (who were excluded). The NHI research database is one of the highest quality databases of its kind in the world, and has been widely used for longitudinal cohort studies [12–17], including our previous research [13,14,17].

The NHRI encrypts all data to protect patient privacy. This database provides patient identification numbers, birth dates, sex, names of medical institutions where care was given, diagnostic codes according to the International Classification of Diseases, 9th Revision, Clinical Modification (ICD-9-CM), prescription use, procedure codes, health care costs, dates of admission and discharge, death dates, outpatient and inpatient claims data, and related information. All data of each individual patient is interlinked through the patient’s unique identification number.

### Study cohort

**Fig 1:**
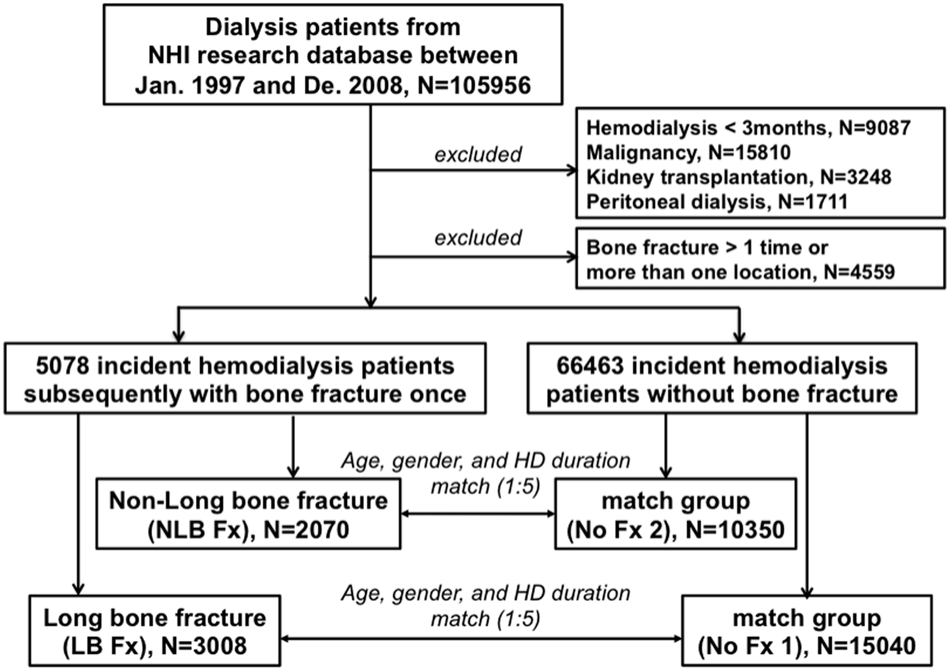
Flowchart of enrolled incident hemodialysis patients.

This study received prior approval from the Ethics Committee and Human Subjects Institutional Review Board of Tzu Chi Hospital, Hualien (TCH IRB No. 101–126). All dialysis patients from the NHIRD and the Registry of Catastrophic Illnesses were enrolled from January 1, 1997 to December 31, 2008. Subjects were excluded if they had malignancies (n = 15810), received dialysis for less than three months (n = 9087), received kidney transplantation (n = 3248), received peritoneal dialysis (n = 1711), or had more than one fracture over follow-up or fractures site at more than one location, to avoid the impact of different kind of fractures (n = 4559) (Fig. 1).

The resulting HD patients were assigned to three groups: incident HD patients subsequently with LB Fx (n = 3008), incident HD patients subsequently with NLB Fx (n = 2070), and incident HD patients with no bone fractures (n = 66463). Finally, cases and controls were enrolled in a 1:5 ratio (with matching by age, sex, and duration of HD) for the following 3 comparisons: *(i)* LB Fx (n = 3008) *vs*. no bone fracture (No Fx-1, n = 15040); *(ii)* NLB Fx (n = 2070) *vs*. no bone fracture (No Fx-2, n = 10350); and *(iii)* LB Fx (n = 3008) *vs*. NLB Fx (n = 2070). All included LB Fx and NLB Fx patients were diagnosed with these conditions before January 2006, so that 3 years of follow-up data were available. The study time of follow-up began from the onset of fracture. To avoid confounding from the effect of HD, each subject in each No Fx group (No Fx-1 and No Fx-2) was matched with a patient in the LB Fx or NLB Fx group that had the same duration of HD between the initiation of hemodialysis and the time of fracture. For example, the No Fx groups are matched on duration of HD (defined as initiation of hemodialysis to time of fracture in fracture patients) and then followed for another 3 years after that. Therefore, if 2 years was the duration of HD, the follow-up for a non-fracture patient would be from years 3–5 after initiation of HD. The diagnosis of a fracture was confirmed in three outpatient visits or one hospitalization. The long bones were the femur, tibia, and fibula of the legs; the humerus, radius, and ulna of the arms; metacarpals and metatarsals of the hands and feet; phalanges of the fingers and toes, and the clavicle. All other bones were classified as non-long bones. (S1 Table)

### Measurements of mortality and atherosclerotic complications

The end points were mortality, stroke, CAD (defined as acute myocardial infarction, old myocardial infarction, angina pectoris, subacute ischemic heart disease, and chronic ischemic heart disease), ACS (acute myocardial infarction and subacute ischemic heart disease), and peripheral arterial occlusive disease (PAOD).

### ICD-9 codes

The ICD-9 codes were determined by patient identification numbers in the NHI databases. The ICD-9-CM codes were: acute myocardial infarction (410), other acute and subacute forms of ischemic heart disease (411), old myocardial infarction (412), angina pectoris (413), other forms of chronic ischemic heart disease (414), COPD (491–494, 496, 510), ESRD (585), diabetes (250.X), hypertension (401.X–405.X), hyperlipidemia (272.X), hemorrhagic stroke (430–432.X), ischemic stroke (433.X–438.X), malignant diseases (140.X–208.X), PAOD (440–444, 447, 451–453, 557), fracture of clavicle (810), fracture of humerus (812), fracture of ulna or radius (813), fracture of phalanx of hand (816), fracture of femur (820–821), fracture of tibia and fibula (823), fracture of phalanx of foot (826), fracture of nasal bone, maxillary bone, or skull(802–803), fracture of spine (805–806), fracture of rib (807), fracture of pelvis (808), fracture of scapula (811), fracture of carpal (814) or metacarpal (815), fracture of patella (822), fracture of ankle (824).

### Statistical analysis

The recorded demographic data included sex, age, and presence of previous comorbid diseases. Patients were divided into three subgroups based on age (≤45, 46 to 64, and ≥65 years-old). The chi-square test was used to compare demographic characteristics of the groups. Multivariate Cox proportional hazards regression models were used to assess the hazard ratios (HRs) and 95% confidence intervals (CIs). SAS statistical software (SAS System for Windows, version 9.1.3; SAS Institute, Cary, NC, U.S.A.) was used for statistical analysis. All statistical tests were 2-sided and a *p*-value less than 0.05 was considered statistically significant.

## Results

### Characteristics of study population

Table 1 shows the baseline characteristics of the study subjects. The percentages of incident HD patients with diabetes, hypertension, and COPD were significantly higher in the LB Fx and NLB Fx groups than in the corresponding no fracture groups. Additionally, the percentage of HD patients with hyperlipidemia was significantly lower in the LB Fx group than in the NLB Fx group and the corresponding no fracture group. Although these are statistically significant, they are likely not clinically meaningful differences.

**Table 1:** Baseline characteristics of enrolled incident hemodialysis patients.

Variable		*LB Fx* N = 3008	*No Fx-1* N = 15040		*NLB Fx* N = 2070	*No Fx-2* N = 10350		*NLB Fx* N = 2070	*LB Fx* N = 3008	
		N(%)	N(%)	*P*	N(%)	N(%)	*P*	N(%)	N(%)	*P*
**Sex**	Female	1815(60.3)	9075(60.3)	1	1272(61.4)	6360(61.4)	1	1272(61.4)	1815(60.3)	0.426
	Male	1193(39.7)	5965(39.7)		798(38.6)	3990(38.6)		798(38.6)	1193(39.7)	
**Age (years)**	≤45	298(9.9)	1490(9.9)	1	252(12.2)	1260(12.2)	1	252(12.2)	298(9.9)	0.002
	46–64	1117(37.1)	5585(37.1)		817(39.5)	4085(39.5)		817(39.5)	1117(37.1)	
	65≥	1593(53.0)	7965(53.0)		1001(48.4)	5005(48.4)		1001(48.4)	1593(53.0)	
**Diabetes**		498(16.6)	2020(13.4)	<0.001	369(17.8)	1399(13.5)	<0.001	369(17.8)	498(16.6)	0.237
**Hypertension**		2475(82.3)	11949(79.4)	<0.001	1709(82.6)	8234(79.6)	0.002	1709(82.6)	2475(82.3)	0.797
**Hyperlipidemia**		838(27.9)	4753(31.6)	<0.001	651(31.4)	3329(32.2)	0.525	651(31.4)	838(27.9)	0.006
**COPD**		795(26.4)	3187(21.2)	<0.001	517(25.0)	2068(20.0)	<0.001	517(25.0)	795(26.4)	0.245

COPD, chronic obstructive pulmonary disease; LB Fx, long bone fracture; No Fx-1, no fracture group matched with the LB Fx group; NLB Fx, non-long bone fracture; No Fx-2, no fracture group matched with the NLB Fx group.

### Cox proportional hazards analysis of long-term outcomes of incident HD patients

After demographic and co-morbidity adjustments, the LB Fx and NLB Fx groups had significantly higher overall mortality (HR = 1.59, *p* < 0.001 and HR = 1.52, *p* < 0.001, respectively) and higher risk for strokes (HR = 1.09, *p* = 0.028 and HR = 1.19, *p* < 0.001, respectively) than the matched no fracture groups (Table 2). Additionally, the NLB Fx group had significantly higher risks of CAD (HR = 1.13, *p* = 0.003) and PAOD (HR = 1.41, *p* < 0.001) than the no fracture group. Importantly, the NLB Fx group also had significantly higher risks of CAD (HR = 1.13, *p* = 0.012) and PAOD (HR = 1.27, *p* = 0.011) than the LB Fx group.

**Table 2:** Cox proportional hazards model for outcomes in incident hemodialysis patients subsequently with long bone fracture, non-long bone fracture, and no fracture.

Variable	*LB Fx* N = 3008	*No Fx 1* N = 15040			*NLB Fx* N = 2070	*No Fx 2* N = 10350			*NLB Fx* N = 2070	*LB Fx* N = 3008		
	N (%)	N (%)	HR	*P*	N (%)	N (%)	HR	*P*	N (%)	N (%)	HR	*P*
**Mortality**	1045 (34.7)	3501 (23.3)	1.59	<0.001	660 (31.9)	2298 (22.2)	1.52	<0.001	660 (31.9)	1045 (34.7)	0.94	0.196
**Stroke**	823 (27.4)	3930 (26.1)	1.09	0.028	603 (29.1)	2619 (25.3)	1.19	<0.001	603 (29.1)	823 (27.4)	1.08	0.157
**CAD**	922 (30.7)	4675 (31.1)	0.99	0.898	707 (34.2)	3204 (31.0)	1.13	0.003	707 (34.2)	922 (30.7)	1.13	0.012
**ACS**	290 (9.6)	1624 (10.8)	0.92	0.181	221 (10.7)	1105 (10.7)	1.03	0.687	221 (10.7)	286 (9.5)	1.12	0.206
**PAOD**	236 (7.8)	1177 (7.8)	1.06	0.382	210(10.1)	778 (7.5)	1.41	<0.001	210 (10.1)	236 (7.8)	1.27	0.011

ACS, acute coronary syndrome; CAD, coronary artery disease; HR, hazard ratio; PAOD, peripheral arterial occlusive disease; LB Fx, long bone fracture; No Fx-1, no fracture group matched with the LB Fx group; NLB Fx, non-long bone fracture; No Fx-2, no fracture group matched with the NLB Fx group.

Adjustments were made for age, sex, diabetes, hypertension, hyperlipidemia, and COPD.

### Mortality in LB Fx and NLB Fx patients with adjustment for demographic and clinical parameters

Table 3 shows the results of a multivariate Cox proportional hazards analysis of the risk for overall mortality. Older age was an independent risk factor for mortality in incidental HD patients. Males have a higher risk of mortality than females in those with LB fracture or without fractures. Additionally, hyperlipidemia was associated with reduced mortality in incident HD patients.

**Table 3:** Cox proportional hazards models for mortality in incident hemodialysis patients subsequently with long bone fracture, non-long bone fracture, and no fracture.

	*Mortality*					
Variable	*LB Fx vs*. *No Fx 1*		*NLB Fx vs*. *No Fx 2*		*NLB FX vs*. *LB Fx*	
	HR (95% CI)	*P*	HR (95% CI)	*P*	HR (95% CI)	*P*
**Group**	1.59 (1.48–1.70)	<0.001	1.52 (1.39–1.66)	<0.001	0.94 (0.85–1.03)	0.196
**Age (years)**						
≤ 45	Reference		Reference		Reference	
46–64	2.67 (2.24–3.19)	<0.001	2.46 (2.03–2.99)	<0.001	2.44 (1.89–3.15)	<0.001
≥ 65	5.09 (4.29–6.05)	<0.001	4.89 (4.05–5.89)	<0.001	4.09 (3.19–5.25)	<0.001
**Sex**						
Female	Reference		Reference		Reference	
Male	1.09 (1.03–1.16)	0.004	1.08 (1.00–1.17)	0.052	1.07 (0.97–1.18)	0.176
**Comorbidity**						
Diabetes	1.03 (0.95–1.13)	0.435	1.03 (0.93–1.15)	0.534	1.12 (0.99–1.27)	0.072
Hypertension	1.01 (0.94–1.09)	0.797	1.03 (0.94–1.13)	0.574	0.88 (0.76–1.00)	0.053
Hyperlipidemia	0.86 (0.80–0.92)	<0.001	0.85 (0.78–0.92)	<0.001	0.89 (0.78–0.99)	0.038
COPD	1.03 (0.96–1.10)	0.466	1.03 (0.95–1.13)	0.43	1.07 (0.96–1.19)	0.201

COPD, chronic obstructive pulmonary disease; LB Fx, long bone fracture; No Fx-1, no fracture group matched with the LB Fx group; NLB Fx, non-long bone fracture; No Fx-2, no fracture group matched with the NLB Fx group.

Adjustments were made for age, sex, diabetes, hypertension, hyperlipidemia, and COPD.

### Stroke in LB Fx and NLB Fx patients with adjustment for demographic and clinical parameters

Table 4 shows the results of a multivariate Cox proportional hazards analysis of the risk for stroke. Incidental HD patients who were male, older, had diabetes, hypertension, hyperlipidemia, or COPD had increased risks for stroke. The risk of stroke was similar in the LB Fx and NLB Fx groups.

**Table 4:** Cox proportional hazards models for stroke in incident hemodialysis patients subsequently with long bone fracture, non-long bone fracture, and no fracture.

	*Stroke*					
Variable	*LB Fx vs. No Fx 1*		*NLB Fx vs. No Fx 2*		*NLB FX vs. LB Fx*	
	HR (95% CI)	*P*	HR (95% CI)	*P*	HR (95% CI)	*P*
**Group**	1.09 (1.01–1.17)	0.028	1.19 (1.09–1.30)	<0.001	1.08 (0.97–1.20)	0.157
**Age (years)**						
≤ 45	Reference		Reference		Reference	
46–64	2.53 (2.16–2.97)	<0.001	2.58 (2.16–3.07)	<0.001	2.71 (2.06–3.56)	<0.001
≥ 65	4.00 (3.42–4.68)	<0.001	4.01 (3.38–4.76)	<0.001	3.70 (2.83–4.84)	<0.001
**Sex**						
Female	Reference		Reference		Reference	
Male	1.10 (1.04–1.17)	0.001	1.10 (1.02–1.18)	0.012	1.04 (0.93–1.16)	0.477
**Comorbidity**						
Diabetes	1.11 (1.03–1.20)	0.007	1.15 (1.05–1.26)	0.003	1.11 (0.98–1.27)	0.111
Hypertension	1.92 (1.75–2.11)	<0.001	1.86 (1.66–2.09)	<0.001	1.96 (1.64–2.35)	<0.001
Hyperlipidemia	1.14 (1.07–1.21)	<0.001	1.15 (1.07–1.24)	<0.001	1.23 (1.10–1.38)	<0.001
COPD	1.08 (1.01–1.15)	0.025	1.11 (1.03–1.21)	0.011	1.08 (0.96–1.21)	0.220

COPD, chronic obstructive pulmonary disease; LB Fx, long bone fracture; No Fx-1, no fracture group matched with the LB Fx group; NLB Fx, non-long bone fracture; No Fx-2, no fracture group matched with the NLB Fx group.

Adjustments were made for age, sex, diabetes, hypertension, hyperlipidemia, and COPD.

### Cox proportional analyses of the risk of cardiovascular diseases in incident HD patients subsequently with LB Fx and NLB Fx

#### NLB Fx patients have higher risks of CAD and PAOD than no Fx patients

Tables 5–7 show the results of multivariate Cox proportional analyses of the risk for 3 major cardiovascular diseases, CAD, ACS, and PAOD. Older age, diabetes, hypertension, and hyperlipidemia were independent risk factors for CAD and PAOD in the model with the NLB Fx and no fracture groups.

**Table 5:** Cox proportional hazards models for coronary artery disease (CAD) in incident Hemodialysis patients subsequently with long bone fracture, non-long bone fracture, and no fracture.

	*CAD*					
Variable	*LB Fx vs. No Fx 1*		*NLB Fx vs. No Fx 2*		*NLB FX vs. LB Fx*	
	HR (95% CI)	*P*	HR (95% CI)	*P*	HR (95% CI)	*P*
**Group**	0.99 (0.93–1.07)	0.898	1.13 (1.04–1.23)	0.003	1.13 (1.03–1.25)	0.012
**Age (years)**						
≤ 45	Reference		Reference		Reference	
46–64	2.00 (1.77–2.27)	<0.001	1.94 (1.70–2.22)	<0.001	1.46 (1.20–1.78)	<0.001
≥ 65	2.46 (2.17–2.78)	<0.001	2.33 (2.04–2.66)	<0.001	1.79 (1.47–2.18)	<0.001
**Sex**						
Female	Reference		Reference		Reference	
Male	1.06 (1.00–1.12)	0.045	1.03 (0.97–1.10)	0.35	0.99 (0.89–1.09)	0.812
**Comorbidity**						
Diabetes	1.15 (1.07–1.23)	<0.001	1.19 (1.09–1.29)	<0.001	1.10 (0.97–1.24)	0.126
Hypertension	2.36 (2.15–2.59)	<0.001	2.26 (2.02–2.52)	<0.001	2.69 (2.22–3.25)	<0.001
Hyperlipidemia	1.52 (1.44–1.61)	<0.001	1.56 (1.46–1.67)	<0.001	1.70 (1.53–1.88)	<0.001
COPD	1.32 (1.24–1.40)	<0.001	1.29 (1.20–1.38)	<0.001	1.28 (1.15–1.42)	<0.001

COPD, chronic obstructive pulmonary disease; LB Fx, long bone fracture; No Fx-1, no fracture group matched with the LB Fx group; NLB Fx, non-long bone fracture; No Fx-2, no fracture group matched with the NLB Fx group.

Adjustments were made for age, sex, diabetes, hypertension, hyperlipidemia, and COPD.

**Table 6:** Cox proportional hazards models for acute coronary syndrome (ACS) in incident hemodialysis patients subsequently with long bone fracture, non-long bone fracture, and no fracture.

	*ACS*					
Variable	*LB Fx vs. No Fx 1*		*NLB Fx vs. No Fx 2*		*NLB Fx vs. LB Fx*	
	HR (95% CI)	*P*	HR (95% CI)	*P*	HR (95% CI)	*P*
**Group**	0.92 (0.81–1.04)	0.181	1.03 (0.89–1.19)	0.687	1.12 (0.94–1.34)	0.206
**Age (years)**						
≤ 45	Reference		Reference		Reference	
46–64	2.10 (1.67–2.64)	<0.001	2.11 (1.65–2.70)	<0.001	1.99 (1.33–2.96)	0.001
≥ 65	2.93 (2.34–3.67)	<0.001	2.84 (2.22–3.62)	<0.001	2.39 (1.61–3.54)	<0.001
**Sex**						
Female	Reference		Reference		Reference	
Male	1.12 (1.02–1.23)	0.016	1.14 (1.02–1.28)	0.02	1.15 (0.96–1.38)	0.141
**Comorbidity**						
Diabetes	1.04 (0.92–1.18)	0.489	0.99 (0.86–1.15)	0.923	1.04 (0.83–1.28)	0.738
Hypertension	1.90 (1.63–2.21)	<0.001	1.91 (1.58–2.29)	<0.001	2.65 (1.86–3.77)	<0.001
Hyperlipidemia	1.44 (1.31–1.59)	<0.001	1.51 (1.35–1.68)	<0.001	1.53 (1.27–1.83)	<0.001
COPD	1.08 (0.97–1.19)	0.177	1.14 (1.00–1.29)	0.046	1.06 (0.87–1.29)	0.563

COPD, chronic obstructive pulmonary disease; LB Fx, long bone fracture; No Fx-1, no fracture group matched with the LB Fx group; NLB Fx, non-long bone fracture; No Fx-2, no fracture group matched with the NLB Fx group.

Adjustments were made for age, sex, diabetes, hypertension, hyperlipidemia, and COPD.

**Table 7:** Cox proportional hazards models for peripheral arterial occlusive disease (PAOD) in incident hemodialysis patients subsequently with long bone fracture, non-long bone fracture and no fracture.

	*PAOD*					
Variable	*LB Fx vs. No Fx 1*		*NLB Fx vs. No Fx 2*		*NLB FX vs. LB Fx*	
	HR (95% CI)	*P*	HR (95% CI)	*P*	HR (95% CI)	*P*
**Group**	1.06 (0.93–1.23)	0.382	1.41 (1.21–1.64)	<0.001	1.27 (1.06–1.54)	0.011
**Age (years)**						
≤ 45	Reference		Reference		Reference	
46–64	1.34 (1.08–1.65)	0.007	1.27 (1.02–1.60)	0.035	1.28 (0.90–1.81)	0.174
≥ 65	1.45 (1.18–1.78)	<0.001	1.27 (1.01–1.59)	0.042	1.44 (1.01–2.03)	0.042
**Sex**						
Female	Reference		Reference		Reference	
Male	1.03 (0.92–1.15)	0.612	0.95 (0.83–1.09)	0.474	1.16 (0.96–1.41)	0.126
**Comorbidity**						
Diabetes	1.25 (1.09–1.43)	0.001	1.25 (1.07–1.47)	0.006	1.17 (0.93–1.48)	0.175
Hypertension	1.47 (1.25–1.73)	<0.001	1.55 (1.27–1.90)	<0.001	1.65 (1.20–2.26)	0.002
Hyperlipidemia	1.63 (1.46–1.82)	<0.001	1.63 (1.43–1.85)	<0.001	1.75 (1.44–2.12)	<0.001
COPD	1.10 (0.97–1.24)	0.138	1.14 (0.99–1.33)	0.075	1.09 (0.89–1.35)	0.402

COPD, chronic obstructive pulmonary disease; LB Fx, long bone fracture; No Fx-1, no fracture group matched with the LB Fx group; NLB Fx, non-long bone fracture; No Fx-2, no fracture group matched with the NLB Fx group.

Adjustments were made for age, sex, diabetes, hypertension, hyperlipidemia, and COPD.

### Different cardiovascular event rate between LB Fx and NLB Fx

CAD and PAOD were more common in patients with NLB Fx than patients with LB Fx. Also, hypertension, and hyperlipidemia were independent risk factors for CAD and PAOD in the model with the NLB Fx and LB Fx groups.

### Outcome events in patients with LB Fx and NLB Fx: multivariate Cox regression model

**Fig 2:**
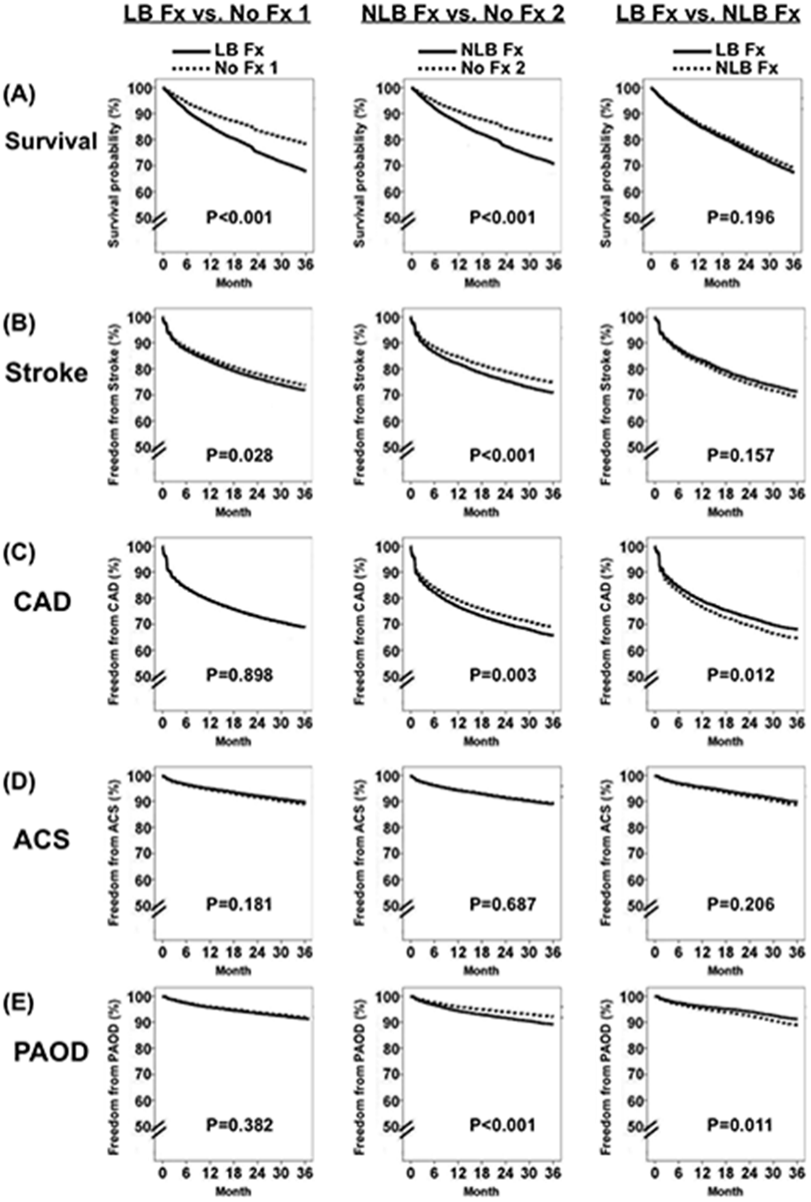
Survival probability and freedom from atherosclerotic complications in incident hemodialysis patients subsequently with long bone fracture (LB Fx), non-long bone fracture (NLB Fx), and without fracture (No Fx) based on the Cox regression model. (A) Survival. (B) Stroke. (C) Coronary artery disease (CAD). (D) Acute coronary syndrome (ACS). (E) Peripheral arterial occlusive disease (PAOD).

Fig. 2 shows survival probability and freedom from atherosclerotic complications of incident HD patients subsequently with LB Fx, NLB Fx, and no fracture groups. These results show that incident HD patients subsequently with LB Fx had increased risks of mortality and stroke compared to incident HD patients *without fractures*, and that incident HD patients subsequently with NLB Fx had increased risks of mortality, stroke, CAD, and PAOD compared to incident HD patients without fractures. Additionally, incident HD patients subsequently with NLB Fx had higher risks of CAD and PAOD than incident HD patients subsequently with LB Fx.

## Discussion

The present study is the first to compare the mortality and atherosclerotic complications of incident HD patients subsequently with LB Fxs, NLB Fxs, and without fractures. Our study had two major findings. First, the rates of mortality and stroke were significantly higher in incident HD patients subsequently with either type of bone fracture than in matched HD patients without fractures. Second, incident HD patients subsequently with NLB Fxs had significantly higher risks of CAD and PAOD than incident HD patients subsequently with LB Fxs or without fractures.

Our finding that incident HD patients subsequently with bone fractures (LB Fx or NLB Fx) had significantly higher mortality than matched HD patients without fractures is consistent with several previous observational studies which showed that the mortality rate was higher in HD patient with hip fractures [6,8], and all kinds of fractures [7,10]. Moreover, our results showed age was the strongest independent risk factor for mortality. In particular, incident HD patients who are more than 65 years old have a ~5-fold higher risk of mortality than with patients who are less than 45 years old. Besides, a large observational study also showed that elderly HD patients with hip fractures had a high mortality rate (16–20%) at one month after fracture [6]. Our results also indicated an inverse association between hyperlipidemia and mortality. This result is in contrast to studies of general populations, but similar to other studies of dialysis patients [18,19]. On the other hand, several randomized trials, including 4-D, AURORA, and SHARP, demonstrated that statin therapy decreased cholesterol levels in HD patients with hypercholesterolemia, but did not affect mortality or cardiovascular outcomes of these patients [20–22].

No previous studies have evaluated the risk of atherosclerotic complications in HD patients subsequently with LB Fxs, although some studies demonstrated that cardiovascular diseases were the major causes of mortality in dialysis patients with hip fractures [6,8]. The results of our study indicate that incident HD patients subsequently with LB Fxs had increased risk of stroke compared to incident HD patients without fractures. Additionally, there was no significant difference in risk of CAD, ACS, and PAOD between incident HD patients subsequently with LB Fxs and incident HD patients without fractures.

No previous studies have examined the risk of atherosclerotic complications in incident HD patients subsequently with NLB Fxs, although a large observational study of HD patients with fractures showed that vertebral fracture was the second most common type of fracture and was the fracture associated with the highest mortality, rate of hospitalization, and duration of hospitalization [7]. This observational study indicated the clinical importance of vertebral fractures in HD patients. Our study was the first to examine the risk of atherosclerotic complications in incident HD patients subsequently with NLB Fxs, and demonstrated that such patients have increased risks of stroke, CAD, and PAOD in incident HD patients relative to incident HD patients without fractures. Additionally, comparison of the NLB Fx and LB Fx groups indicated that the risks of CAD and PAOD were significantly higher in the NLB Fx group. The reasons for the differences in atherosclerotic complications in the LB Fx and NLB Fx groups are unknown. One possibility is that they are due to the different effects of secondary hyperparathyroidism on cortical and trabecular bones [23,24].

The main strength of this study is that the Taiwan NHI research database is one of the largest and most reliable databases of its kind, and has been widely used in many previous studies [12–17]. Nevertheless, there were some limitations in this study. First, subjects’ privacy is protected by the NHI research database, so we could not obtain body height, weight, education, occupation, information on personal habits (physical activity, lifestyle, smoking, or alcohol consumption), disease severity, or family history. Second, laboratory data could not be obtained, so we did not know the adequacy of dialysis, level of intact parathyroid hormone, and basic biochemical data. Third, the causes of death were not available from the NHIRD, so we could not compare the causes of death in patients with and without bone fractures. Fourth, the competing risk of death was not considered in any of the analyses and adjustment for multiple comparisons was not done which might increase the likelihood of false findings. Despite these limitations, this is the first nationwide and population-based analysis to compare the risks of mortality and different components of atherosclerotic complications in HD patients with and without bone fractures.

In conclusion, this cohort study showed that incident HD patients subsequently with bone fractures (LB Fx or NLB Fx) had significantly higher rates of mortality and stroke than incident HD patients without fractures. In addition, incident HD patients subsequently with NLB Fxs had a significantly increased risk of CAD and PAOD than incident HD patients subsequently with LB Fx or without fractures. Thus, incident HD patients subsequently with bone fractures, especially those who are elderly, should be more closely monitored for a high mortality and potential atherosclerotic complications.

## Supporting Information

S1 Table Number and percentage of different bone fractures. (DOC)
