# Supplementary material for: Increased Risks of Mortality and Atherosclerotic Complications in Incident Hemodialysis Patients Subsequently with Bone Fractures: A Nationwide Case-Matched Cohort Study
Source: PLoS One. 2015 Apr 13;10(4):e0121705. doi: 10.1371/journal.pone.0121705 (PMC4395346; doi:10.1371/journal.pone.0121705)
Supplement: S1 Table — (DOC) [file pone.0121705.s001.doc]

**Supplement Table.** Number and percentage of different bone fractures

| **Types of Bone Fractures** | Number | % |
| --- | --- | --- |
| **Long Bone Fractures** | 3008 | 100 |
| Femur | 1423 | 47.31 |
| Ulna or radius | 557 | 18.52 |
| Humerus | 408 | 13.56 |
| Clavicle | 217 | 7.21 |
| Phalanx of hand | 206 | 6.85 |
| Tibia and fibula | 197 | 6.55 |
|  |  |  |
| **Non-Long Bone Fractures (N=2070)** | 2070 | 100 |
| Spine | 1018 | 49.18 |
| Rib | 449 | 21.69 |
| Ankle | 293 | 14.16 |
| Pelvis | 158 | 7.63 |
| Nasal bone, maxillary or skull | 73 | 3.53 |
| Carpal | 51 | 2.46 |
| Scapula | 28 | 1.35 |
